# 

**Published:** 1943

**Authors:** 


### The Bristol Medico-Chirurgical Journal

A JOURNAL OF THE MEDICAL SCIENCES FOR THE WEST OF ENGLAND AND SOUTH WALES

PUBLISHED UNDER THE AUSPICES OF

*THE BRISTOL MEDICO-CHIRURGICAL SOCIETY*

EDITOR

J. A. NIXON, C.M.G., M.D., F.R.C.P.

WITH WHOM ARE ASSOCIATED

A. E. ILES, O.B.E., M.B., B.S., F.R.C.S.

A. RENDLE SHORT, M.D., B.S., B.Sc., F.R.C.S.

W. MELVILLE CAPPER, M.B., B.S., F.R.C.S.

E. WATSON-WILLIAMS, M.C., Ch.M., F.R.C.S., Assistant Editor

"Scire est nescire, nisi id me

Scire alius sciret"

*VOL. LX*

BRISTOL: J. W. ARROWSMITH LTD.

### Contents of Vol. LX.

| | Page |
|---|---|
| The Future of Health and Hospital Services from an Architect's Point of View. By Charles E. Elcock, F.R.I.B.A. .. .. .. .. | 1 |
| Social and Economic Conditions and the Incidence of Rheumatic Heart Disease. By G. H. Daniel, D.Phil. .. .. .. .. | 13 |
| Reviews of Books .. .. .. .. .. .. .. .. .. | 27 |
| Editorial Notes .. .. .. .. .. .. .. .. .. | 32 |
| Obituaries .. .. .. .. .. .. .. .. .. .. | 33 |
| Medical Library of the University of Bristol .. .. .. .. | 37 |
| The One Hundred and Seventy-seventh List of Books, Etc. .. | 38 |
| Publications Received .. .. .. .. .. .. .. .. | 40 |
| Local Medical Notes—Examination Results .. .. .. .. | 41 |

### The Medical Library of the University of Bristol

WITH WHICH IS INCORPORATED

**The Library of the Bristol Medico-Chirurgical Society**

---

*The following donations have been received since the publication of the list in the last issue.*

| | | |
|---|---|---|
| British Postgraduate Medical School (1) | (unbound) | 3 volumes. |
| Brompton Hospital (2) .. .. .. .. .. | | 1 volume. |
| Mrs. E. Fawcett (3) .. .. .. .. .. | | 10 volumes. |
| Dr. Josef Heller (4) .. .. .. .. .. | | 2 volumes. |
| Michell Clarke Bequest (5) .. .. .. .. | | 6 volumes. |
| Mrs. Muirhead (6) .. .. .. .. .. | | 1 volume. |
| Sheffield University (7) .. .. .. .. .. | | 2 volumes. |
| Universities' Federation for Animal Welfare (8) .. | | 1 volume. |
| Dr. H. Secker Walker (9) .. .. .. .. | | 5 volumes. |
| General Medical Council (10) .. .. .. .. | | 1 volume. |

Unbound periodicals have also been received from Dr. S. B. Adams, Professor E. W. Hey Groves, Dr. T. Milling, Professor C. Bruce Perry, Dr. J. Odery Symes, Messrs. John Wright & Sons Ltd.

### THE ONE HUNDRED AND SEVENTY-SEVENTH LIST OF BOOKS.

The figures in round brackets refer to the figures after the names of the donors. The books to which no such figures have been attached have either been bought from the Library Fund or received through the *Journal*.

| | | |
|---|---|---|
| **Bevington, S. M.** | *Nursing Life and Discipline* | 1943 |
| **Beyer, H.** | *Die Operationen am Ohr* (4) | 1932 |
| **Bowman, A. K.** | *The Life and Teaching of Sir William McEwen* | 1942 |
| **Boyd, Wm.** | *Textbook of Pathology* 4th Ed. | 1943 |
| **Charras, M.** | *The Royal Pharmacopoeia, Galenical and Chymical* | 1678 |
| **Clendenning, L.** | *Source book of Medical History* | 1942 |
| **Dieckmann, W. J.** | *Toxemias of Pregnancy* (5) | 1942 |
| *Documents Medicaux* | (3) | |
| **Edridge-Green, F. W.** | *Colour-blindness and colour perception* 2nd Ed. (9) | 1909 |
| *Encyclopaedia Medica* | Volume 15 (1st Supp.) (6) | 1910 |
| **Fisher, R. A.** | *The Design of Experiments* | 1942 |
| **Forkner, C. E.** | *Leukaemia and allied disorders* (5) | 1938 |
| **Fry, K. et al** | *The Dental Treatment of Maxillo-facial Injuries* | 1943 |
| **Gibberd, G. F.** | *A Short Text-book of Midwifery* 3rd Ed. | 1943 |
| **Gibson, W. M.** | *Dental Prosthetics* | 1941 |
| **Glaister, J.** | *Medical Jurisprudence and Toxicology* 7th Ed. | 1942 |
| **Gray, H.** | *Textbook of Anatomy* 28th Ed. | 1942 |
| **Guedel, A. E.** | *Inhalation Anæsthesia* (5) | 1937 |
| **Hadfield, G., and Garrod, J. P.** | *Recent Advances in Pathology* 4th Ed. | 1942 |
| **Hall, I. S.** | *Diseases of the Nose, Throat and Ear* 2nd Ed. | 1941 |
| **Hewer, C. L.** | *Recent Advances in Anæsthesia and Analgesia* 4th Ed. | 1943 |
| **Hill, A. V.** | *Adventures in Biophysics* | 1931 |
| **Horne, W. J. and Cheatle, A. H.** | *Descriptive Catalogue of Museum of 6th Internat. Otolog. Conference of* 1899 (9) | 1900 |
| **Hueper, W. C.** | *Occupational Tumors and Allied Diseases* | 1942 |
| **Hunter, J.** | *Natural History of the Human Teeth* (7) | 1781 |
| **Illingworth, C. F. W.** | *Textbook of Surgical Treatment* | 1943 |
| **Illingworth, C. F. W., and Dick, B. M.** | *A Textbook of Surgical Pathology* 4th Ed. (Rept.) | 1943 |
| **Lamb, F. W.** | *Introduction to Human Experimental Physiology* | 1930 |
| **Langdon-Brown, W., and Hilton, R.** | *Physiological Principles in Treatment* 8th Ed. (5) | 1943 |
| **Lavater, J. C.** | *Essays on Physiognomy* 5 Vols. | 1810 |
| **Lewis T.** | *Diseases of the Heart* 3rd Ed. | 1943 |

**Macintosh, R. R., and Bannister, F. B.** *Essentials of General Anæsthesia* 3rd Ed. 1943

**Marrack, J. R.** .. .. *Food and Planning* .. .. .. .. .. .. .. .. 1943

*Minutes of the General Medical Council.* LXXIX for 1942 .. (10) 1943

**Neal, J. B.** .. .. .. *Encephalitis* .. .. .. .. .. .. .. .. 1942

**Nightingale, F.** .. .. *Notes on Hospitals* .. .. .. .. .. (7) 1863

**Politzer, A.** .. .. *Lehrbuch der Ohrenheilkunde.* 3te Aufl .. (9) 1893

**Seiffert, A.** .. .. .. *Die Operationen an Nase, Mund und Hals* (4) 1936

**Souder, W., and Paffenbarger, G. C.** *Physical Properties of Dental Materials* .. .. .. .. .. .. .. .. 1942

**Syme, J.** .. .. .. *Contributions to the Pathology and Practice of Surgery* .. .. .. .. .. .. .. (9) 1848

**Tandler, J.** .. .. .. *Anatomie des Herzens* .. .. .. .. .. (3) 1913

**Ten Teachers** .. .. *Midwifery* .. .. .. .. .. .. .. .. 7th Ed. 1942

*Vaccine and Serum Therapy* .. .. .. .. .. .. .. .. .. (9) 1935

**Yoffey, J. M.** .. .. *In Memoriam, Edward Fawcett* .. (Pamphlet) 1943

**Wright, J. G.** .. .. *Veterinary Anæsthesia* .. .. .. .. .. (8) 1942

### TRANSACTIONS, REPORTS, JOURNALS, ETC.

Brompton Hospital Reports. Vol. XI .. .. .. .. .. .. .. (2) 1942

Collected Papers of the British Postgraduate Medical School, 1939-42. 3 vols. .. .. .. .. .. .. .. .. .. .. .. .. .. (1) 1942

Dental Board of the United Kingdom Reports .. .. .. .. .. (3)

The Dentists Register .. .. .. .. .. .. .. .. .. .. .. .. 1943

General Medical Council, Minutes, 1942 .. .. .. .. .. .. .. 1943

The Hospitals Yearbook .. .. .. .. .. .. .. .. .. .. .. 1942

The Medical Directory .. .. .. .. .. .. .. .. .. .. .. .. 1943

The Medical Annual .. .. .. .. .. .. .. .. .. .. .. .. 1943

Quarterly Cumulative Index Medicus. Vols. 31 and 32 .. .. .. .. 1942

Quarterly Cumulative Index Medicus. January to March .. .. .. .. 1943

Transactions of the Opthalmological Society. Vol. 61 .. .. .. .. 1941

Transactions of the Ophthalmological Society. Vol. 62 .. .. .. .. 1942

The Yearbook of Radiology .. .. .. .. .. .. .. .. .. (5) 1942

The Yearbook of Eye, Ear, Nose and Throat .. .. .. .. .. (5) 1942

### Publications Received

From The British Pharmacopoeia Commission :

*Sixth Addendum to the British Pharmacopoeia,* 1932–1943.

From Messrs. Cassell & Co. Ltd. :

*Modern Operative Surgery* (Carson's). Volume I. Edited by G. Grey Turner, F.R.C.S. Price 50s. 1943.

*Students Handbook of Surgical Operations* (Treves). Edited by C. P. G. Wakeley, F.R.C.S. Price 12s. 6d. 1943.

From Messrs. H. K. Lewis & Co. Ltd. :

*Guide to F. A. Treatment of Home Guard Casualties.* By T. H. Meek. Price 4s.

*Physical Exercises for Asthma.* Issued by The Asthma Research Council. Price 1s.

From The Oxford University Press (Oxford War Manuals) :

*Ear, Nose and Throat in the Services.* By W. Stevenson. Price 5s.

*Scabies.* By F. Mellanby. Price 5s.

From Messrs. John Wright & Sons Ltd. :

*Electrocardiograms.* Second Edition. N. Jones and E. Noble Chamberlain. Price 5s.

*Infant Feeding in General Practice.* Second Edition. By J. V. Braithwaite. Price 7s. 6d.

*Synopsis of Surgical Anatomy.* Fifth Edition. By A. Lee Macgregor. Price 25s.

*British Journal of Surgery.* Volumn XXX. Nos. 119, 120. Price 12s. 6d. each.

*Symptoms and Signs in Clinical Medicine.* Third Edition. By E. Noble Chamberlain. Price 30s.

### Local Medical Notes

EXAMINATION RESULTS.

**University of Bristol.**—Students of the University have recently passed the following examinations :—

M.B., Ch.B.—*First Examination. Pass :* Barbara Brosnan, R. J. Carey, Suzanne K. R. Clarke, Vera H. M. Dowling, D. N. Fitzgerald, Anne M. French, J. C. Gregg, B. T. Hale, R. B. Handscombe, Pamela I. Hannaford, Ruth E. Hazell, A. H. Levy, June Morgan, Frances W. Parrott, D. B. Peacock, Jenny Pym, Jacqueline I. Shaw, P. J. Speller, B. F. Vaughan, M. P. Walker, Pamela L. C. Watson, Audrey M. Wells, R. H. Wood. *In Chemistry :* Kathleen J. Harrison,† A. J. Lee.†

M.B., Ch.B.—*Second Examination* (*Section I*). *Pass :* J. H. Beatson, W. H. K. Carpenter, M. J. Dunn, W. H. N. Moore, Eleanor B. Webb, Norma M. Whalley, G. Winternitz. (*Section II—Completing the Examination.*) Patricia M. Appleton, W. E. Arnold, Dorothy J. Blake, Kathleen Blott, D. E. Bolt, W. H. K. Carpenter, P. J. Chapman, Elizabeth H. Collis, M. J. Dunn, Pamela M. Farmer, H. L. Jones, M. B. Lennard, J. C. Little, Florence E. Moore, J. H. K. Parker, Hazel M. Passmore, H. S. Paull, C. D. R. Pengelly, Dorothy E. M. Pierce, R. G. G. Taylor, Jean Tregear, K. D. J. Vowles, Kathleen G. Wright.

M.B., Ch.B.—*Final Examination* (*Section I*). *Pass :* R. I. Bodman, M. P. Bourke, (Mrs.) E. M. Carey, (Mrs.) I. M. M. Codd, Margot J. Copland, Margaret P. Drew, J. Eskell, E. C. Hamlyn, W. A. Heaton-Ward, Kathleen C. Iles, Flavia Z. L. B. James, P. A. James, A. S. Jones, Christine M. H. Jones, D. M. M. Jones, M. H. Kerby, (Mrs.) L. M. Leitch, R. Maggs, Mildred I. Pott, R. J. Sandry, F. I. Tovey (1, 2), (Mrs.) M. J. Williams (2), Elizabeth N. R. Wilson.

M.B., Ch.B.—*Final Examination. Pass :* J. E. Beviss, M. P. Bourke, A. G. P. Davies (3), Mercia J. Griffiths, C. H. Gurd, Elisabeth Hodson, (Mrs.) L. M. Leitch, D. E. Olliff, Mildred I. Pott (4), D. A. Sarsfield, S. S. Short (5), T. R. Stephens. *In Group II only :* (Mrs.) M. J. Noakes, Elizabeth N. R. Wilson.

B.D.S.—*First Examination. Pass : In Chemistry :* G. D. Pamely. *In Chemistry and Biology :* L. K. Walden.

B.D.S.—*Second Examination. Pass :* P. J. Blyth, E. J. R. Morgan (6), P. J. Sheldrick, W. Watson.

B.D.S.—*Third Examination. Pass :* A. N. R. Gibson. *In Dental Mechanics, etc. :* J. M. Allen.† *In Dental Anatomy only :* G. A. J. Comyn (7), D. C. Sara, A. F. J. Smith.

B.D.S.—*Final Examination. Pass :* F. G. Lloyd. *In Section II only :* Mrs. M. J. Miller.†

L.D.S.—*First Examination. Pass :* T. C. Hughes, D. A. Pearce.

L.D.S.—*Second Examination. Pass :* A. D. Baxter, L. G. D. Brown, B. L. Evans.

L.D.S.—*Third Examination. Pass :* K. W. Bowry, J. F. V. Woolley. *In Dental Mechanics*, etc. : L. H. Valentine,† *In Dental Anatomy :* T. W. Beer,† Mary P. Hazell.

L.D.S.—*Final Examination. Pass :* R. L. Yendall. *Section II* (*Dental Surgery*) *:* R. D. Hepburn,† N. W. Leech,† L. Murray,† B. N. Neilson,† B. A. Oliver.† *Section I :* J. W. B. Kay, F. E. R. Williams.

**University of Cambridge.**—M.B.—*Final Examination. Part II* (Principles and Practice Physics, Pathology and Pharmacology) : *Pass :* Helen M. Comely.

**University of Oxford.**—B.M.—*Second Examination* (Medicine, Surgery and Midwifery) : *Pass :* Sheila M. Tangye.

**Society of Apothecaries, London.**—*Pre-Medical and Primary Examinations for L.M.S.S.A. : Pass :* A. J. E. Richards,* (Chemistry, Biology).

**Royal College of Surgeons of England.**—*Final Examination, L.D.S. Pass : Part I :* F. M. Warren. *Part II :* F. M. Warren, S. Phillips.

**Conjoint Board.**—L.R.C.P., M.R.C.S.—*First Examination. Pass :* Janet Baker (8), Elizabeth Bryant (9, 10), (Mrs.) E. M. Carey (8), P. J. Chapman (9, 10), (Mrs.) I. M. M. Codd (8), Margot J. Copland (8), Jyotikana Datta (8), (Mrs.) M. J. Noakes (8), D. Purnell (9, 10), Dorothy M. Reece (9, 10), Margaret E. J. Richards (10), A. J. F. Saxton (8), Rae H. Scott (9, 10), T. Straton (9, 10), Rosemary V. Styles (9, 10), W. B. Tindall (9, 10).

L.R.C.P., M.R.C.S.—*Final Examination. Pass :* Janet Baker (11, 12), M. P. Bourke (11), F. J. Goddard (13),† Elisabeth Hodson (11, 12, 13), A. J. R. Hudson (14),† W. G. Hunt (12, 14),† D. J. Jones (12, 14),† P. H. Keppich (12), E. J. Perks (13),†* Mildred I. Pott (12), J. T. Rendle-Short (12), A. J. F. Saxton (11), G. Steiner (12),†* I. F. R. Sutherland (11, 13, 14), Dorothy H. Thorne (13, 14),† (Mrs.) Dorothy Willoughby (11, 12).

* Former Student.
† Completing Examination.
(1) With Distinction in Materia Medica, Pharmacy, etc.
(2) With Distinction in Pathology and Bacteriology.
(3) With Distinction in Forensic Medicine and Toxicology.
(4) With Distinction in Public Health.
(5) With Distinction in Surgery.
(6) With Distinction in Anatomy.
(7) With Distinction in Dental Anatomy.
(8) Pharmacology and Materia Medica.
(9) Anatomy. (10) Physiology. (11) Pathology.
(12) Midwifery. (13) Surgery. (14) Medicine.

### Bristol Medico-Chirurgical Society

### President.

C. CORFIELD, M.D. Durh.

### President-Elect.

### Committee.

*Ex-Officio* { J. A. NIXON, *C.M.G.*, B.A., M.D. Cantab., F.R.C.P. (Editor of *Journal*).

(Vacant), (Hon. Librarian).

*Elected.*

L. A. MOORE, M.B., Ch.B. Brist. (Ex-President).
R. C. CLARKE, *O.B.E.*, M.B., Ch.B.Brist.
H. H. CARLETON, M.A., M.D., B.Ch.Oxon.
A. L. TAYLOR, M.D. Lond.
P. S. MARTIN, *M.C.*
T. MILLING, M.B., Ch.B. Belf.
W. V. WOOD, *M.C.*, B.A. Cantab.
H. L. SHEPHERD, M.B., Ch.M. Brist.
W. A. JACKMAN, F.R.C.S.

### Honorary Secretary.

R. V. COOKE, Ch.M.

### Honorary Treasurer.

A. E. ILES, *O.B.E.*, M.B., B.S. Lond., F.R.C.S.

### University Library Sub-Committee.

A. R. SHORT, B.Sc., M.D. Lond., F.R.C.S.
C. BRUCE PERRY, M.D. Brist., F.R.C.P.
H. J. DREW SMYTHE, *M.C.*, M.S., M.D. Lond., F.R.C.S., F.R.C.O.G.

Medical Practitioners wishing to join the Society are requested to communicate with the Hon. Secretary, Mr. R. V. COOKE, Failand Lodge, Guthrie Road, Clifton. The Annual Subscription is £1 1s., payable to Hon. Treasurer.

---

*Extracts from Journal By-laws.*

4.—The *Journal* shall be delivered free to Subscribers and also to Members of the Society whose subscriptions are not in arrear.

6.—The Annual Subscription to the *Journal* is 9/–, payable to Hon. Treasurer.

---

Communications intended for insertion in the *Journal* should be sent to the Editor, Dr. J. A. NIXON, 7 Lansdown Place, Clifton, Bristol.

Books for Review and Exchange Journals should be sent to the Assistant Editor, *Bristol Medico-Chirurgical Journal*, Medical Library, The University, Bristol. Reviews should be returned with the books to Mr. E. WATSON-WILLIAMS, 65 Pembroke Road, Bristol 8.

Communications referring to the delivery of the *Journal* should be sent to the Hon. Treasurer, Mr. A. E. ILES, 87 Pembroke Road, Bristol 8.

Volumes of the *Journal* can be bound upon application to J. W. ARROWSMITH LTD., Quay Street, Bristol, price on request. The inclusive index for Volumes I—L may be obtained, price 3/– (postage extra).

### Bristol Medico-Chirurgical Society

### PAST PRESIDENTS

1874—75. FREDERICK BRITTAN, M.D., B.S. Dub.
1875—76. ROBERT WILLIAM COE.
1876—77. SAMUEL MARTYN, M.D. Ed.
1877—78. AUGUSTIN PRICHARD, M.D. Berlin.
1878—79. HENRY EDWARD FRIPP, M.D. Würzburg.
1879—80. WILLIAM MICHELL CLARKE.
1880—81. JOSEPH GRIFFITHS SWAYNE, M.D. Lond.
1881—82. EDWARD LONG FOX, M.D. Oxon.
1882—83. JAMES GEORGE DAVEY, M.D. St. And.
1883—84. WILLIAM JOHNSTONE FYFFE, M.D., B.A. Dub.
1884—85. GEORGE FORSTER BURDER, M.D. Aberd.
1885—86. WILLIAM HENRY SPENCER, M.D., M.A. Cantab.
1886—87. FRANCIS POOLE LANSDOWN.
1887—88. LEMUEL MATTHEWS GRIFFITHS.
1888—89. ROBERT SHINGLETON SMITH, M.D., B.Sc. Lond.
1889—90. NELSON CONGREVE DOBSON, Ch.M. Brist.
1890—91. SAMUEL HENRY SWAYNE.
1891—92. FRANCIS RICHARDSON CROSS, M.B. Lond., LL.D. Brist.
1892—93. EDWARD MARKHAM SKERRITT, M.D., B.S., B.A. Lond.
1893—94. JAMES GREIG SMITH, M.B., C.M., M.A. Aberd., F.R.S.E.
1894—95. ALFRED JAMES HARRISON, M.B. Lond.
1895—96. ARTHUR WILLIAM PRICHARD.
1896—97. ALFRED EDWARD AUST LAWRENCE, M.D., C.M. Aberd.
1897—98. JOHN EDWARD SHAW, M.B., C.M. Ed.
1898—99. ROBERT ROXBURGH, M.B. Ed.
1899—00. WILLIAM HENRY HARSANT.
1900—01. DAVID SAMUEL DAVIES, M.D. Lond.
1901—02. BARCLAY JOSIAH BARON, M.B., C.M. Ed.
1902—03. GEORGE MUNRO SMITH, M.D. Brist.
1903—04. JAMES PAUL BUSH, C.M.G., C.B.E., Ch.M. Brist
1904—05. REGINALD EAGER, M.D. Lond.
1905—06. JOHN DACRE.
1906—07. JAMES TAYLOR.
1907—08. HENRY WALDO, M.D., C.M. Aberd.
1908—09. JOHN MICHELL CLARKE, M.D., M.A. Cantab.
1909—10. JAMES SWAIN, C.B., C.B.E., M.D., M.S. Lond.
1910—11. BERTRAM MITFORD HERON ROGERS, M.D., B.Ch., B.A. Oxon.
1911—12. CHARLES ALEXANDER MORTON.
1912—13. WALTER CARLESS SWAYNE, M.D., B.S. Lond. and Brist.
1913—14. PATRICK WATSON-WILLIAMS, M.D. Lond., M.D. (Hon. Causa) Brist.
1914—15. WILLIAM HARRY CHRISTOPHER NEWNHAM, M.A., M.B. Cantab.
1915—16. GEORGE PARKER, M.A., M.D. Cantab., LL.D. Brist.
1916—17. FRANCIS HENRY EDGEWORTH, M.A., M.D. Cantab., D.Sc. Lond.
1917—18. FREDERICK LACE.
1918—19. ROBERT GUTHRIE POOLE LANSDOWN, M.D., B.S. Durh.
1919—20. I. WALKER HALL, M.D., Ch.B. Vict.
1920—21. LEOPOLD ERNEST VALENTINE EVERY-CLAYTON, M.D. Lond.
1921—22. CYRIL HUTCHINSON WALKER, B.A., M.B. Cantab.
1922—23. JOHN ALEXANDER NIXON, C.M.G., B.A., M.D. Cantab.
1923—24. JOHN LACY FIRTH, M.D., M.S. Lond.
1924—25. JOHN ODERY SYMES, M.D. Lond.
1925—26. THOMAS CARWARDINE, M.S. Lond.
1926—27. ARTHUR LAUNCELOT FLEMMING, M.B., Ch.B. Brist.
1927—28. WALTER KENNETH WILLS, M.A., M.B., B.Ch. Cantab.
1928—29. HENRY LAWRENCE ORMEROD, M.D., B.Ch. R.U.I.
1929—30. CHARLES FERRIER WALTERS.
1930—31. DAVID CHARLES RAYNER, Ch.M. Brist.
1931—32. JOHN ROGER CHARLES, M.A., M.D. Cantab.
1932—33. ERNEST WILLIAM HEY GROVES, M.D., M.S., D.Sc., B.Sc. Lond.
1933—34. HERBERT ELWIN HARRIS, B.A., M.B. Cantab.
1934—35. A. RENDLE SHORT, B.Sc., M.D. Lond.
1935—36. ARTHUR ERNEST ILES, O.B.E., M.B., B.S. Lond.
1936—37. RICHARD CHRISTOPHER CLARKE, O.B.E, M.B. Lond.
1937—38. CLIFFORD A. MOORE, M.B., M.S. Lond.
1938—40. LEONARD AUGUSTUS MOORE, M.B., Ch.B. Brist.
1940—41. CHARLES CORFIELD, M.D. Durh., Barr.-at-Law.
1941—42.
1942—43.

### 1943.

### LIST OF SUBSCRIBERS

TO THE

### Bristol Medico-Chirurgical Journal.

### HONORARY MEMBER OF THE SOCIETY.

Swain, James, *C.B.*, *C.B.E.*, M.D., M.S. Lond. .. .. .. Wyndham House, Easton-in-Gordano.

### SUBSCRIBERS.

*Those marked * are Members of the Society.*

| | |
|---|---|
| *Adams, A. W., M.S. Lond. .. .. .. .. | Elton House, Clifton, Bristol 8. |
| Adams, J. B .. .. .. .. .. .. .. .. | Kent Lodge, Eastbourne. |
| *Adams, S. B., Ph.D., M.B., Ch.B. Brist. .. | Clapton Hill, near Portishead. |
| *Aldwinckle, Helen M., M.B., Ch.B. Brist. .. | 23 Rockland Road, Downend, Bristol. |
| *Alexander, A. J. P., M.D. Belf. .. .. .. | Winsley Sanatorium, near Bath. |
| *Alexander, D. A., M.B., Ch.B. Vict. .. .. | 112 Pembroke Road, Clifton, Bristol 8. |
| Alexander, G. L., B.A., M.B., B.Ch. Cantab. | West African Medical Service, Laura, via Accra, Gold Coast. |
| *Alford, A. F., M.B., Ch.B. Brist. .. .. | Board of Education, Belgrave Square London, S.W.1. |
| Alford, H. J., M.D. Lond. .. .. .. .. | 4 Park Terrace, Taunton. |
| Ashton, L. P., M.B., Ch.B. Brist. .. .. .. | Kapsowar, P.O. Eldoret, Kenya, East Africa |
| *Aubrey, T., M.B. Lond. .. .. .. .. .. | Bitton, near Bristol. |
| *Bain, W. .. .. .. .. .. .. .. .. .. | 1 Greville Road, Southville, Bristol 3. |
| *Baker, Lily A., M.D., B.Ch., B.A.O., R.U.I. | 23 Pembroke Road, Clifton, Bristol 8. |
| Ball, Mrs. J. J. .. .. .. .. .. .. .. | St. Leonard's Vicarage, Redfield, Bristol 5. |
| *Barber, M. C., M.B., Ch.B. Brist. .. .. .. | Ingledene, High Street, Staple Hill, Bristol. |
| *Barbour, Lieut.-Col. R. F., R.A.M.C. .. | 13 Old Sneed Road, Stoke Bishop, Bristol. |
| *Barker, G. H., M.D., B.Sc. Lond. .. .. .. | 124 Redland Road, Bristol 6. |
| *Bates, R. M. .. .. .. .. .. .. .. .. | Purdown House, Stapleton, Bristol 5. |
| *Baxter, Ursula, M.B., Ch.B. Brist. .. .. | 167 Wells Road, Knowle, Bristol 4. |
| *Baxter, V. T., M.B., Ch.B. .. .. .. .. | 167 Wells Road, Knowle, Bristol 4. |
| *Beatson, D. H., M.B., Ch.B. Brist. .. .. | 8 Richmond Park Road, Clifton, Bristol 8. |
| *Bell, R. J. Irving .. .. .. .. .. .. | 5a Oakfield Road, Clifton, Bristol 8. |
| *Bergin, F. G. .. .. .. .. .. .. .. .. | 31 Oakfield Road, Clifton, Bristol 8. |
| *Berry, R. J. A., Prof., M.D., C.M. Edin. .. | 9 Derby Street, Ormskirk, Lancs. |
| *Birrell, J. A., M.D., B.S. Lond. .. .. .. | 1 Victoria Square, Bristol 8. |
| Blackett, J. F., M.D., B.S. Lond. .. .. .. | Hamsterley, Bloomfield Park, Bath. |
| Bletchly, G. Playne, M.B. Lond. .. .. | c/o Lloyds Bank, Nailsworth, Glos. |
| *Bodman, A. P., M.B., Ch.B. .. .. .. .. | 5 Hurle Crescent, Clifton, Bristol 8. |
| *Bodman, F. H., M.D., Ch.B. Brist. .. .. .. | 2 Redland Court Road, Bristol 6. |
| Bolt, R. F. .. .. .. .. .. .. .. .. .. | 70 Great Russell Street, London, W.C.1. |
| *Boulton, B. J., M.B., Ch.B. Brist. .. .. | Ham Green Hospital, Pill, near Bristol. |
| *Bown, Naomi J., M.B., Ch.B. Brist. .. .. | The Corner, Nailsea, Somerset. |
| *Bradbeer, E. G., M.B., Ch.B. Brist. .. .. | 13 Percival Road, Bristol 8. |
| Brasher, C. W. J., M.D. .. .. .. .. | Murivance, Nether Stowey, Somerset. |
| *Brinckman, Winifred P., M.B., Ch.B. Brist. | 2 Clifton Park, Bristol 8. |
| *Britton, R. B., M.B., B.S. Lond. .. .. .. | 33a Whiteladies Road, Clifton, Bristol 8. |
| *Brocklehurst, R. J., M.A., D.M. Oxon. .. | The University, Bristol 8. |
| Brown, J. E., M.B., Ch.B. Brist. .. .. .. | Coulthurst Villa, Brives Road, Maraval, Trinidad, B.W.I. |
| Bryan, C. .. .. .. .. .. .. .. .. .. | 44 Nevil Road, Bishopston, Bristol 7. |
| *Burges, R. .. .. .. .. .. .. .. .. .. | Druid's Mead, Stoke Bishop, Bristol 9. |
| *Bush, G. B., M.B., Ch.B. Brist. .. .. .. | Litfield House, Clifton, Bristol 8. |

| | |
|---|---|
| *CAIRNS, F. J. | Devon House, Whitehall Road, Bristol 5. |
| CAMERON, MARGARET, M.B., Ch.B. | 1 Dean Lane, Bristol 3. |
| *CAPPER, W. M., M.B., B.S. Lond. | Litfield House, Clifton, Bristol. |
| *CAREY, R. S., *O.B.E.*, B.A. Cantab. | 502 Bath Road, Brislington, Bristol 4. |
| *CARLETON, H. H., M.A., M.D., B.Ch. Oxon. | 1 Lansdown Road, Clifton, Bristol 8. |
| *CASSON, E., M.D., Ch.B. Brist. | Mount Pleasant, Clevedon. |
| *CHAMBERS, E. R. | 9 Clifton Park, Clifton, Bristol 8. |
| *CHARLES, J. R., M.A., M.D. Cantab. | Litfield House, Clifton Down, Bristol 8. |
| *CHITTY, H., M.S. Lond. | Naish House, Clapton-in-Gordano. |
| *CHRISTOFFERSON, P. E., M.B., Ch.B. Brist. | 14 Oakfield Road, Clifton, Bristol 8. |
| *CLAREMONT, L. E., M.D.S. Brist. | 5 Rodney Place, Clifton, Bristol 8. |
| *CLARK, DENNIS O. | 168 Milton Road, Bristol Road, Weston-super-Mare. |
| *CLARKE, R. C., *O.B.E.*, M.B., Ch.B. Brist. | Harley Lodge, Clifton Park, Bristol 8. |
| *COATES, H. | Notts County Mental Hospital, Radcliffe-on-Trent. |
| *COCHRANE, J. H. | 11 Bryant's Hill, St. George, Bristol 5. |
| *COLLINGWOOD, F. C., M.B., Ch.B. Brist. | c/o C.M.O., British Airways, 20 College Road, Clifton 8. |
| *COOKE, ELIAZBETH M., M.D. Lond. | 53 Queen's Court, Clifton 8. |
| *COOKE, R. V., Ch.M. Brist. | Litfield House, Bristol 8. |
| *COOKSON, R. G. | Clifton College, Erdiston, Bude. |
| COOMBS, N. C. | Romaldkirk, Barnard Castle, Co. Durham. |
| *COOPER, W. R. | 13 Westfield Park, Redland, Bristol 6. |
| *CORFIELD, C., M.D. Durh. | 5 Kensington Place, Clifton, Bristol 8. |
| *CORNER, BERYL, M.D., B.S. Lond. | 3 Elton House, Rodney Place, Bristol 8. |
| *COSSHAM, W. L., M.B., Ch.B. Brist. | 3 Claremont Road, Bishopston, Bristol 7. |
| *COURTNEY, R. M., M.B., Ch.B. Glasg. | Manor House, Southmead Road, Bristol |
| *COXON, BEATRICE | 16 Richmond Hill, Clifton, Bristol 8. |
| *CRAIG, ANNE A., M.D., B.S. Lond. | 35 Queen's Court, Clifton, Bristol 8. |
| *CROOK, B. A., M.B., Ch.B. Brist. | The Laurels, Timsbury, near Bath. |
| CROOT, H. J., M.B., B.S. Lond. | Royal Infirmary, Bristol 2. |
| *CROSSMAN, F. W., M.B. Durh. | White's Hill, Hambrook, near Bristol. |
| *DATTA, S. S., M.D. Brist. | Snowdon House, Fishponds, Bristol. |
| *DAVIES, E. D. D. | Standish House, Stonehouse, Glos. |
| *DAVIES, J. N. P., M.B., Ch.B. | Physiology Department, The University, Bristol. |
| *DELANEY, N., M.B., Ch.B. Manch. | The Meade, Bishopsworth, Somerset. |
| *DICKSON, W. A., M.D. St. And. | Standish House, Stonehouse, Glos. |
| *DISNEY, M., M.D. Lond. | On Active Service. |
| *DIX, C. | 14 Mortimer Road, Clifton, Bristol 8. |
| *DIXON, T. B. | R.N. Sick Quarters, Dover. |
| *DODD, GRANTHAM, M.B., B.S. | 42 Woodstock Road, Bristol 6. |
| *DUERDEN, J. R., M.B., Ch.B. Brist. | 4 Cecil Road, Bristol 8. |
| *DUTTON, HILDA R. | 405 Stapleton Road, Bristol 5. |
| *DYER, W. P. | West View, Westbury-on-Trym, Bristol. |
| *EGLINTON, J. H. C. | Street, Somerset. |
| ELLIOTT, F. P., M.B., C.M. Aberd. | 113 Grove Road, Walthamstow, London, E. 17. |
| *ELLIOTT, W. H. A., M.B., B.S. Lond. | 29 Old Sneed Avenue, Stoke Bishop, Bristol 9. |
| *ELLIS, W. T. | Tregenna House, Shirehampton. |
| *EMERY, J. L. | Childrens' Hospital, Bristol 2. |
| *EVANS, G. M., M.B., Ch.B. Brist. | 117 Ashley Road, Bristol. |
| *EYRE-BROOK, A. L., M.B., M.S. Lond. | The Cottage, Portishead. |
| *FALLON, Col. J. | 35 Henleaze Gardens, Henleaze, Bristol. |
| *FAWN, G. F. | 6 Oakfield Road, Clifton, Bristol 8. |
| *FELLS, G. R., M.B., Ch.B. Brist. | 19 Southmead Road, Bristol. |
| *FELLS, R. R., M.B., B.S. Lond. | 17 Mortimer Road, Clifton, Bristol 8. |
| *FENELEY, G. L., M.B., Ch.B. Brist. | Englewood, Falcondale Road, Westbury-on-Trym. |

| | |
|---|---|
| *FINZEL, H. F. M., M.D. Brist. | Nare House, Birchwood Road, St. Anne's Park, Bristol 4. |
| *FISHER, A. C., M.D. Brist. | Roan Antelope Mine, N. Rhodesia. |
| *FITZGIBBON, G. M., M.D. Lond. | 75 Pembroke Road, Clifton, Bristol 8. |
| *FITZGIBBON, L. PRISCILLA, M.B., B.S. Lond. | 75 Pembroke Road, Clifton, Bristol 8. |
| *FORRESTER-BROWN, MAUD, M.D., M.S. Lond. | 22 Combe Park, Bath. |
| *FOSS, G. L., M.B., B.Ch. Cantab. | Cloud's Hill House, St. George, Bristol 5. |
| *FOX, F. E., B.A. Cantab. | Brislington House, Brislington, Bristol 4. |
| *FRASER, A. D., M.A., M.B., Ch.B. Glasg. | 4 Alexandra Road, Clifton, Bristol 8. |
| *FRASER, D., M.B., Ch.B. Edin. | Westgate Dursley, Glos. |
| *FULLER, H. L. | 9 Gay Street, Bath. |
| | |
| *GARDEN, R. R., M.A., M.B., B.Ch. Aberd. | 9 Harley Place, Clifton, Bristol 8. |
| GARLAND, E. B., M.B., C.M. Edin. | 114a Hampton Road, Redland, Bristol 6. |
| *GERRISH, NORMAN, M.B., Ch.B. Brist. | 2 Station Road, Keynsham, Som. |
| *GIBBS, J. R. | 48 Codrington Road, Bishopston, Bristol 7. |
| *GORHAM, A. P., M.B., Ch.B. Brist. | 53 Westbury Road, Westbury-on-Trym. |
| *GORNALL, W. A., M.D. Brist. | Ulvik, Nailsea, near Bristol. |
| *GREEN, S. B., M.B. Lond. | The Black Wicket, Westbury-on-Trym. |
| *GREENLAW, MADGE E., M.B., Ch.B. Brist. | 206 Redland Road, Bristol 6. |
| GRIFFITHS, CORNELIUS A. | 35 Newport Road, Cardiff. |
| *GROVES, E. W. HEY, M.D., M.S., D.Sc. Lond. | 25 Victoria Square, Clifton, Bristol 8. |
| | |
| *HAM, C. I., M.B., Ch.B.Brist. | 89 Stonebridge Park, Eastville, Bristol. |
| *HARRIS, H. E., M.A., M.B., B.Ch. Cantab. | 13 Lansdown Place, Clifton, Bristol 8. |
| *HARTLEY, GRACE, M.B., B.S. Lond. | 49 Queen's Court, Clifton, Bristol 8. |
| *HEALES, J. N., M.B., Ch.B. Brist. | Holmcroft, Street, Somerset. |
| *HECTOR, F., M.D. Lond. | 1 Victoria Square, Clifton, Bristol 8. |
| *HEMPHILL, R., M.D. Dub. | 90 Cleeve Hill, Downend, near Bristol. |
| *HENDERSON, JAMES, M.B., Ch.B. Aberd. | The City Sanatorium, Yardley Road, Birmingham. |
| *HERAPATH, C. E. K., *M.C.*, M.D.Lond. | Ormlie, Clifton Down, Bristol 8. |
| *HEWER, Professor T. F., M.D. Brist. | Canynge Hall, Bristol 8. |
| *HILL, WINIFRED, M.B., Ch.B. Brist. | c/o Lloyd's Bank, Ilford. |
| *HOFFMAN, H. LOVELL, B.A., M.B., B.Ch. Cantab. | R.N. Hospital, Invergordon. |
| *HOLLAND, W. A. L. | 10 Brunswick Square, Bristol. |
| *HOOKER, J. A., Colonel, M.B., Ch.B.Brist. | 16 St. Edyth's Road, Sea Mills 9. |
| *HOSKEN, J. G. F. | Uley, Glos. |
| HOSPITAL, BRISTOL GENERAL (*Secretary*, T. W. GREGG). | |
| *HOUGHTON, LYDIA M., M.B., B.S. Lond. | 74 Church Road, Redfield, Bristol 5. |
| *HOWELL, T. E. | 6 Richmond Hill, Bristol 8. |
| *HUGHES, F. W. TERRELL, M.B., Ch.B. Brist. | Chew Magna, Somerset. |
| *HUGHES, MARGUERITE G., M.B., Ch.B. Brist. | Walnut Tree Farm, East Dundry, Som. |
| *HUNTER, F. S. | c/o Messrs. J. Wright & Sons Ltd., Publishers, 28 Orchard Street Bristol 1. |
| *HYATT, ANNIE W., M.B., B.S. Lond. | Merrymead, Shepton Mallet, Som. |
| | |
| *ILES, ANNA, M.B., Ch.B. Brist. | 87 Pembroke Road, Clifton, Bristol 8. |
| *ILES, A. E., *O.B.E.*, M.B., B.S. Lond. | 87 Pembroke Road, Clifton, Bristol 8. |
| INFIRMARY, BRISTOL ROYAL (*Secretary*, E. C. SMITH). | |
| INGLE, C. DURBAN | Somerton, Somerset. |
| *ISSERLIN, B. | 2 College Road, Bristol 8. |
| | |
| *JACKMAN, W. A., *T.D.*, M.B., Ch.B. Brist. | 15 Mortimer Road, Clifton, Bristol 8. |
| *JAMES, APRIL D., M.B., Ch.B. Brist. | (Address unknown.) |
| *JAMES, J. A., M.D. Lond. | Litfield House, Clifton Down, Bristol 8. |
| *JAMES, J. A., Senr. | Dusk, Elmlea Avenue, Stoke Bishop, Brist 1, 9. |
| *JENKINS, F. G., M.B., Ch.B. Brist. | 51 Redcliff Hill, Bristol 1. |
| *JENKINS, R. D. | The Copper Beeches, Almondsbury, near Bristol. |
| *JENKINS, P. K., M.B., Ch.B. | R.A.M.C. |

| | |
|---|---|
| *JOHNSON, R. G., M.B. Lond. | 119 Redland Road, Bristol 6. |
| *JOLL, C. A., M.D., M.S. Lond. | 64 Harley Street, London, W.1. |
| *JONES, MEGAN E. | Royal Infirmary, Bristol 2. |
| *JORDAN, L. R., M.B., Ch.B. Brist. | National Temperance Hospital, Hampstead Road, N.W.1. |
| *JOSCELYNE, P. C., M.B., Ch.B. Brist. | |
| *KEMM, N. F., M.B., B.S. Lond. | 200 Redland Road, Durdham Park, Bristol, 6. |
| *KERSLEY, G. D., M.D. | 6 The Circus, Bath. |
| KING, E. F. | 2 Upper Wimpole Street, London, W.1. |
| *KINGSTON, S. H., M.B., Ch.B. Brist. | 3 Whiteladies Road, Clifton, Bristol 8. |
| *KYLE, H. G., M.A., M.D., B.Ch. Oxon. | 31 Westbury Road, Bristol. |
| *LACE, F | 5 Gay Street, Bath. |
| LALONDE, T. P., M.B., Ch.B. Brist. | Wykeham House, Romsey, Hants. |
| *LEES, E. LEONARD, M.D., C.M. Edin. | 28 Downs Park West, Bristol 6. |
| *LEGAT, R. EDDOWES, M.B., C.M. Edin. | Hampden, St. Briavels, Glos. |
| *LEWIS, F. J., M.B., Ch.B. Brist. | Canynge Hall, Bristol 8. |
| *LINTON, MARION S., M.B. Lond. | 1 Brecon Road, Henleaze, Bristol. |
| *LISTER, A. E. J., M.B., B.S. Lond. | 86 Pembroke Road, Clifton, Bristol 8. |
| *LOGAN, H. B. | Elmwood, Aberdeen Road, Cotham, Bristol 6. |
| *LOXTON, S. D., M.B., Ch.B. | |
| *LUCAS, J. E. | c/o 132 York Road, Bristol 3. |
| *LUCAS, J. J. S., M.D. Lond. | 186 Wells Road, Knowle, Bristol 4. |
| *LUCAS, J. V. | 186 Wells Road, Knowle, Bristol 4. |
| *LUCAS R. P., M.B., Ch.B. Brist. | 15 Edgecumbe Road, Redland Bristol 6. |
| *MCKENZIE, W. G., *M.C.* | 11 Bath Road, Reading. |
| *MACLACHLAN, A. M., M.B., Ch.B. Glasg. | 65 Redcliff Hill, Bristol 1. |
| *MARSHALL, A. H., M.B., Ch.B. Brist. | 281 Canford Lane, Bristol. |
| *MARWOOD, S. F., M.D., B.S. | Troy House, Kingswood, Bristol. |
| *MCLANNAHAN, J. G. | The Mount, Stonehouse. |
| *MACLAREN, CATHERINE J., M.B., Ch.B. Edin. | Hereford House, Clifton Down. |
| MACLEAN, SIR EWEN J., M.D., C.M. Edin. | 12 Park Place, Cardiff |
| *MACLEOD, G., M.A. Glas., M.D., M.B. | Wargrave, Clevedon, Somerset. |
| *MARSHALL, A. H., M.B., Ch.B. Brist. | 281. Canford Lane, Bristol. |
| MARSHALL, A. L., M.B., B.A. Cantab. | Laxfield, Woodbridge, Suffolk. |
| *MARTIN, P. S., *M.C.* | Victoria Quadrant, Weston-super-Mare. |
| *MATHESON, N. R., M.B., Ch.B. | 37 Abbey Road, Westbury-on-Trym. |
| MIALL, C. L'O | Elm Hayes, Paulton, near Bristol. |
| *MILLING, T., M.B., Ch.B. Belf. | 1 Vicarage Road, Southville, Bristol 3. |
| *MOORE, C. A., M.B., M.S. Lond. | South Lodge, Kingsweston Road, Henbury Bristol. |
| *MOORE, L. A., M.B., Ch.B. Brist. | Hillsborough, Cotham Park, Bristol 6. |
| *MOORE, R. H., M.B., Ch.B. Brist. | White Cross Court, Wells Road, Bristol 4. |
| *MORGANS, C. C., M.B., Ch.B. Cantab. | 65 Lower Redland Road, Bristol 6. |
| *MORRIS, L. N., M.B., Ch.B. Brist. | 13 Belgrave Road, Clifton, Bristol 8. |
| *MUNDY, G. S., M.B., B.Ch. Brist. | Homewood, Coombe Dingle, Bristol. |
| *MYLES, Q. St. L., M.A., B.M., B.Ch. Oxon. | 7 Apsley Road, Clifton, Bristol 8. |
| *NICHOLSON-LAILEY, J. R. | Elmfield House, South Road, Taunton. |
| *NIXON, DOREEN, M.B., B.S. Lond. | 7 Lansdown Place, Clifton, Bristol 8. |
| *NIXON, J. A., *C.M.G.*, M.D. Cantab. | 7 Lansdown Place. Clifton, Bristol 8. |
| *NOBLE, J. I., M.B., Ch.B. Liverp. | 102 Pembroke Road, Clifton, Bristol 8. |
| *NORMAN, R. M., M.D. Brist. | Highwood, Stapleton Park, Bristol. |
| *NOTT, WINIFRED, M.B., Ch.B. Brist. | Fassiefern, 8 Rylestone Grove, Stoke Bishop, Bristol 9. |
| *O'DONOHOE, MONICA A., M.B., Ch.B. | 1 Beaconsfield Road, Bristol 8. |
| *ORMEROD, G. L. M.A., M.B., B.Ch. Cantab | 2 Henleaze Road, Westbury-on-Trym, Bristol. |
| *ORR-EWING, H. J., *M.C.*, M.D. Lond. | 1 Beaufort Buildings, Clifton, Bristol 8. |
| *PALIN, A. G., B.M., B.Ch. Oxon. | Litfield House, Clifton Down, Bristol 8. |
| *PARKES, J. A., M.B., Ch.B. Liverp. | 8 South Road, Redland, Bristol 6. |

| | |
|---|---|
| *PARKES, P. W. J. | 164 Filton Avenue, Bristol 7. |
| PARKINSON, Lt.-Col. G. S., *D.S.O.*, R.A.M.C. | London School of Hygiene and Tropical Medicine, Keppel Street, London, W.C. 1. |
| *PARRY, R. H., M.B., B.S.Lond. | Public Health Offices, Bristol 1. |
| PARSONS, Sir J. H., M.B., B.S., B.Sc. Lond. | 54 Queen Anne Street, London, W. |
| *PAUL, R. G. | 23 Pembroke Road, Bristol 8. |
| *PAULI, C. H. | Pagan's Hill Farm, Chew Stoke. |
| *PERRY, B., M.D., Ch.B.Brist. | Canynge Hall, Clifton, Bristol 8. |
| PHELPS, P. C., M.B., Ch.B. | 23 St. Alban's Road, Bristol 6. |
| *PHILLIPS, P., M.B., Ch.B. Brist. | Southmead Hospital, Bristol. |
| *PIRRIE, A. L., M.B., Ch.B. Glasg. | 528 Kensington Hill, Bristol. |
| *POCOCK, J. A., M.A., M.B., B.Ch. Cantab. | 75 Drayton Gardens, London, S.W. 10. |
| PORTER, C. P. | 28 Mill Street, Kidderminster. |
| *POTTER, MABEL F., M.B., Ch.B. Brist. | 79 Pembroke Road, Clifton, Bristol 8. |
| *POWELL, H. F., M.D. Brux. | Ellenborough Park, Weston-super-Mare. |
| *PRICE, C. H. G., M.B., Ch.B. Brist. | Claverham House, Claverham, Yatton. |
| *PRICE, N. L., M.B., Ch.B. Brist. | Litfield House, Clifton Down, Bristol 8. |
| *PRIDIE, K. H., M.B., B.S. Lond. | 58 St. John's Road, Clifton, Bristol 8. |
| *PROWSE, D. C., M.B., Ch.B. Brist. | Wigmore House, Thornbury, near Bristol. |
| *RANKIN, P. C., M.B., Ch.B. Glasg. | Lawrence Hill House, Bristol. |
| *RAYNER, D. C., Ch.M. Brist. | 9 Lansdown Place, Clifton, Bristol 8. |
| *ROBERTS, J. A. F., M.A. Cantab., M.B., Ch.B., D.Sc. Edin. | Stoke Park Colony, Bristol. |
| *ROBERTSON, D., M.B., Ch.B. Edin. | 2 Knowle Road, Knowle, Bristol 4. |
| *ROBERTSON, L. G., M.B., Ch.B. | 162 Redland Road, Bristol 6. |
| *ROGERS, H., M.D. Brist. | 91 Old Park Ridings, Grange Park, N.21. |
| *ROLFE, R., B.A., M.B., B.C. Cantab. | Park House, St. Andrew's Road, Avonmouth. |
| *ROWLANDS, R. D. | Connaught Military Hospital, Knaphill, Surrey. |
| *ROWLEY, A. T. | 13 Walliscote Road, Weston-super-Mare. |
| *RUDOLF, MAJOR G. DE M. | R.A.M.C. |
| *RYAN, P. B., M.B., Ch.B. | 43 Nevil Road, Bishopston, Bristol. |
| *SAMPSON, O. E. L., M.B., Ch.B. Brist. | 77 Stackpool Road, Bristol 3. |
| *SCARFF, G., M.B., Ch.B. Edin. | 83 Pembroke Road, Clifton, Bristol 8. |
| *SHEPHERD, H. L., M.B., Ch.M. Brist. | 79 Pembroke Road, Clifton, Bristol 8. |
| *SHORT, A. R., B.Sc., M.D., B.S.Lond. | 69 Pembroke Road, Clifton, Bristol 8. |
| *SMITH, T. W., M.D. Lond. | 6 Oakley, Claverton Down, Bath. |
| *SMYTHE, H. J. D., *M.C.*, M.D., M.S Lond. | On Active Service. |
| *SNAWDON, J. W. E., M.B., Ch.B., B.D.S. | Litfield House, Clifton, Bristol 8. |
| *SOLTAU, H. K. V., M.D. Lond. | 19 The Avenue, Clifton, Bristol 8. |
| *SOMERS, MARY A. E. | 2 Ashcombe Road, Weston-super-Mare. |
| *SPOOR, A., M.B., B.Ch. Cantab. | The Hydro, Bristol 1. |
| *STEPHEN, R. J. | 92 Redland Road, Bristol 6. |
| *STROVER, H. W. M., *O.B.E.*, M.B., Ch.B. Aberd. | 90 Redland Road, Bristol 6. |
| *STRUTHERS, A. J., M.B., Ch.B. Glasg. | 100 Church Road, Redfield, Bristol 5. |
| *STRUTHERS, GEO., M.B., Ch.B. Glasgow | 100 Church Road, Bristol 5. |
| *SUTTON, G. E. F., M.D. Lond. | 1 Pembroke Road, Clifton, Bristol 8. |
| *SYMES, J. O., M.D. Lond. | 6 Pembroke Vale, Clifton, Bristol 8. |
| *TALLACK, MAJOR R. J. K., R.A.M.C. | 5th (Kenya) Field Amb., A.P.O., E.A. Command. |
| *TASKER, D. G. C., M.B., M.S. Lond. | 9 College Fields, Clifton, Bristol 8. |
| *TAYLOR, A. L., M.D. Lond. | The General Hospital, Bristol 1. |
| TAYLOR, A. L. | The Royal Infirmary Bristol 2. |
| THIN, J. | 54 and 55 South Bridge, Edinburgh. |
| *TODD, A. T., *O.B.E.*, M.D. Edin. | Royal Park House, Clifton, Bristol 8. |
| TRINICK, RICHARD H. | 42 Cherry Valley Gardens, Belfast. |
| TRIPP, DOROTHY M. H. | Mission Hospital, Karim Nagar, Nizam's Dominions, South India. |
| *TRIST, J. R. R., *M.C.* | 120 Henleaze Road, Henleaze. |
| *TRYON, VICTORIA S., M.B., Ch.B. Brist. | 3 Harley Place, Clifton, Bristol 8. |

| | |
|---|---|
| *WAGSTAFFE, E. M., M.B., Ch.B. .. .. .. | Winford Orthopædic Hospital. |
| *WANSBROUGH, T. B., M.B., Ch.B. Brist. .. | 23 Pembroke Road, Clifton, Bristol 8. |
| *WALKER, CYRIL H., B.A., M.B. Cantab. .. | Harewood, Clapton-in-Gordano, Som. |
| *WALTERS, C. F. .. .. .. .. .. .. .. .. | Downover, Walton Down, near Clevedon. |
| WAKE, S. C., M.B., Ch.B. Brist. .. .. .. | "Stanhope," Bideford, N. Devon. |
| *WARD, H. W. .. .. .. .. .. .. .. .. | Chipping Manor, Wotton-under-Edge. |
| *WARREN, R., M.A., M.D. Oxon. .. .. .. | Heathgates, Weston-super-Mare. |
| *WATSON-WILLIAMS, E., *M.C.*, B.A., M.B. Cantab., Ch.M. Brist. | 65 Pembroke Road, Clifton, Bristol 8. |
| WATTS, J. B. V. .. .. .. .. .. .. .. .. | Frantzina's Rust, Barberton, Transvaal, S. Africa. |
| WEDDELL, C. W. .. .. .. .. .. .. .. .. | Boydville, 46 Miller Street, West Melbourne, Australia. |
| *WIGODER, SYLVIA, M.A., M.D., Ph.D. .. .. | General Hospital, Bristol 1. |
| *WILBOND, R. G. .. .. .. .. .. .. .. .. | 76 Chesterfield Road, St. Andrew's Pk., Bristol. |
| *WILLIAMS, I., M.B., Ch.B. Brist. .. .. .. | "Ardrem," Hill View, Henleaze, Bristol. |
| *WILLIAMS, S. R., M.B. Lond. .. .. .. .. | Buckfastleigh, Devon. |
| *WILLWAY, F. W., M.D., M.S. Lond. .. .. | Bristol Royal Infirmary. |
| *WOOD, D. .. .. .. .. .. .. .. .. .. | 105 Pembroke Road, Clifton, Bristol 8. |
| *WOOD, URSULA, M.B., Ch.B. .. .. .. .. | Henley Lodge, Yatton, Som. |
| *WOOD, W. V., *M.C.*, B.A. Cantab. .. .. .. | Henley Lodge, Yatton, Som. |
| *WOODMAN, DOROTHY, M.D. Lond. .. .. .. | Clifton Hill House. |
| *WOOLLEY, W., M.B., Ch.B. Brist. .. .. .. | 239 Cranbrook Road, Redland. |
| *WRIGHT, A. J. M., M.B., B.S. Lond. .. .. | Harley Cottage, Clifton Park, Clifton, Bristol 8. |
| *WRIGHT, WINIFRED M., M.B., B.S. Lond. .. | Merrymead, Shepton Mallet, Som. |
| *ZBALAND, L., M.D. Man. .. .. .. .. .. .. | Brecon Lodge, Westbury-on-Trym, Bristol. |

### *HONORARY VISITING MEMBERS.*

BRIDGES, MAJOR ARTHUR BRODIE HAMILTON, R.A.M.C., *O.B.E.*

COLSON, SURGEON REAR-ADMIRAL HENRY ST. CLAIR, *O.B.E.*, M.D. Lond.

WHITBY, COLONEL LIONEL ERNEST HOWARD, *A.M.S.*, *C.V.O.*, *M.C.*, M.D. Cantab.

WORTHINGTON, COLONEL FRANK, *A.M.S.*, *D.S.O.*, *O.B.E.*, M.B., B.Ch. Cantab.

And their staffs.

